# Oxidative-Induced Angiogenesis Is Modulated by Small Extracellular Vesicle miR-302a-3p Cargo in Retinal Pigment Epithelium Cells

**DOI:** 10.3390/antiox11050818

**Published:** 2022-04-22

**Authors:** Maria Oltra, Miriam Martínez-Santos, María Ybarra, Hugo Rowland, María Muriach, Javier Romero, Javier Sancho-Pelluz, Jorge M. Barcia

**Affiliations:** 1 Neurophysiology and Neurobiology, School of Medicine and Health Sciences, Catholic University of Valencia San Vicente Mártir, 46001 Valencia, Spain; miriam.msantos@ucv.es (M.M.-S.); mariaybarras98@gmail.com (M.Y.); hurow@mail.ucv.es (H.R.); jm.barcia@ucv.es (J.M.B.); 2 Centro de Investigación Translacional San Alberto Magno, Catholic University of Valencia San Vicente Mártir, 46001 Valencia, Spain; 3 Doctoral School, Catholic University of Valencia San Vicente Mártir, 46002 Valencia, Spain; 4 School of Health Sciences, University Jaume I, Av. Vicent Sos Baynat, 12006 Castellón de la Plana, Spain; muriach@med.uji.es; 5 Hospital General de Requena, Hospital de Requena Calle Casablanca, 46340 Valencia, Spain; romero_fragom@gva.es

**Keywords:** microRNAs, small extracellular vesicles, retinal pigment epithelial cells, vasculogenesis

## Abstract

Extracellular vesicles are released from cells under diverse conditions. Widely studied in cancer, they are associated with different diseases playing major roles. Recent reports indicate that oxidative damage promotes the release of small extracellular vesicle (sEVs) from the retinal pigment epithelium (RPE), with an angiogenic outcome and changes in micro-RNA (miRNA) levels. The aim of this study was to determine the role of the miRNA miR-302a-3p, included within RPE-released sEVs, as an angiogenic regulator in cultures of endothelial cells (HUVEC). ARPE-19 cell cultures, treated with H_2_O_2_ to cause an oxidative insult, were transfected with a miR-302a-3p *mimic*. Later, sEVs from the medium were isolated and added into HUVEC or ARPE-19 cultures. sEVs from ARPE-19 cells under oxidative damage presented a decrease of miR-302a-3p levels and exhibited proangiogenic properties. In contrast, sEVs from miR-302a-3p-*mimic* transfected cells resulted in control angiogenic levels. The results herein indicate that miR-302a-3p contained in sEVs can modify VEGFA mRNA expression levels as part of its antiangiogenic features.

## 1. Introduction

Environmental cell conditions can promote the release of extracellular vesicles (EVs) [1,2,3,4]. EVs can be sub-micro-sized, known as microparticles, or nanometric-sized, previously known as exosomes and currently known as small extracellular vesicles (sEVs) [5,6,7]. Normally, sEVs can include several molecules in their cargo, such as proteins, lipids, and genetic material, e.g., micro-RNA (miRNA) [8,9]. Current evidence indicates that sEVs can regulate long-distance organs or neighboring cells and modulate tumor progression and metastasis [10,11,12,13,14]. Moreover, sEVs have been pointed out as potential therapeutic molecules and might be key for early diagnosis on different eye-related disorders [15,16].

Lately, miRNA–non-coding, short (around 22 nucleotides), single-stranded RNA molecules have also been highlighted as potential therapeutic tools and diagnostic markers [17,18,19,20]. miRNAs are able to interact with mRNA strains, inhibiting them, thus reducing protein expression. They are found inside the cell as well as the extracellular medium, including plasma. Circulating miRNAs might travel free in blood plasma or, as mentioned above, as part of the sEVs cargo [21]. This fact has been recently assayed in non-small-cell lung cancer cells by engineering miR-449a as sEVs cargo against the evolution of the disease [22].

It is well described that oxidative insults lead to several responses in many cell types. Concretely, retinal pigment epithelial cells (RPE) are under constant oxidative challenge because of sunlight and high metabolic rate, among others. This circumstance has been pointed out as partially responsible of some proliferative eye-related diseases such as wet age-related macular degeneration (AMD) and diabetic retinopathy (DR) [23]. It has been shown that oxidative stress modifies some VEGF-related miRNAs in ARPE-19 cells, thus promoting angiogenesis [24,25]. In addition, oxidative challenge also promotes excessive sEVs release, which contain some miRNAs—miR-302-a and miR-122—that have been significantly reduced as a consequence of the damage. In contrast, these two miRNAs were unaltered on ARPE-19 cell homogenates [24]. Vascular overproduction is a common trait for different diseases, such as DR, wet AMD, and cancer [26,27,28]. Therefore, neovascularization is one of the most wanted targets to improve the evolution of such diseases [29,30,31].

The miR-302-307 cluster is highly expressed in embryonic stem cells in contrast to differentiated cells and regulates cell cycle [32]. MiR-302 cluster inhibits angiogenesis by targeting VEGFA [33] and has also been involved in the reduction of vascular inflammation according to the binding sequence by interaction with zinc finger protein 91 (ZFP91). ZFP91 activates the hypoxia-induced factor 1α (HIF-1 α), promoting cell proliferation [34,35].

All in all, considering the dramatic H_2_O_2_-induced sEVs release and the paradoxical decreased of miR-302a levels contained on the sEVs [4], we demonstrate herein the vascular influence of the sEVs miR-302a-3p cargo after oxidative challenge on ARPE-19 cells.

## 2. Materials and Methods

### 2.1. Cell Culture

The arising retinal pigment epithelium (ARPE-19) human cell line was obtained from the American Type Culture Collection (ATCC, Manassas, VA, USA). ARPE-19 cells were cultured in Dulbecco’s modified Eagle’s medium/F12 (Invitrogen, Carlsbad, CA, USA), as previously described [24]. Cells were used from 11 to 30 passages. Cells were cultured to 80–90% confluence at a seeding density of 1 × 10^6^ cells/cm^2^. After 48 h of seeding, cells were treated with 600 µM H_2_O_2_ (Scharlau, Barcelona, Spain) and/or 4 mM N-acetylcysteine (NAC; Sigma-Aldrich, St. Louis, MO, USA) for 24 h grown in media supplemented with 1% of exosome-depleted fetal bovine serum (FBS; Thermo Fisher Scientific, Waltham, MA, USA). Cell media and cells were collected and preserved for future experiments.

Human umbilical vein endothelial cells (HUVEC) were isolated from umbilical veins as previously described [36]. HUVEC were grown in endothelial cell media (PromoCell, Heidelberg, Germany) supplemented with 20% FBS, penicillin/streptomycin, and amphotericin at 37 °C and 5% CO_2_.

### 2.2. Cell Viability

Cell viability as mitochondrial activity was measured using 3’-[1-phenylaminocarbonyl-3,4-tetrazolium]-bis(4-methoxy-6-nitro) benze sulfonic acid hydrate (XTT; Cell Proliferation Kit II; Roche, Basel, Switzerland). ARPE-19 cells were seeded at 6 × 10^3^ cells per well in a 96-cell culture well plate for 24 h. Cells were rinsed with PBS twice and then were incubated with 0.3 mg/mL of XTT final solution for 6 h at 37 °C in 5% CO_2_. Mitochondrial activity was measured by fluorescence multiple reader (Victor X5; Perkin Elmer) at 550 nm.

### 2.3. Vasculogenesis Assay

Vasculogenesis was performed in Matrigel (Becton Dickinson, Franklin Lakes, NJ, USA) as previously described [36]. Briefly, HUVEC were seeded 8 × 10^4^ cells per well in a 96 well plate and then treated with ARPE-19 cell media for 5 h. The different conditions were determined by the conditioned medium used: control media, sEVs from control media, sEVs from mimic-miR-302a-3p transfected-control media, sEVs from 600 µM H_2_O_2_ media, sEVs from mimic-miR-302a-3p transfected 600 µM H_2_O_2_ media and 4 mM NAC media. Matrigel was allowed to solidify overnight at 37 °C. Pictures were taken with an Olympus CKX41 inverted microscope (Olympus, Tokyo, Japan) and recorded by an Olympus DP74 digital camera (Olympus, Tokyo, Japan). Angiogenesis Analyzer for ImageJ was used to analyze the total tube length of the taken images.

### 2.4. sEVs Isolation

sEVs isolation was performed using the Total Exosome Isolation Reagent from cell culture media (Thermo Fisher Scientific, Waltham, MA, USA). The 2 mL of cell media was centrifuged at 2000× *g* for 30 min to remove cells and debris. The supernatant containing the cell-free culture media was mixed with 1 mL of Total Exosome Isolation reagent and incubated overnight at 4 °C. After incubation, the sample was centrifuged at 10,000× *g* for 1 h at 4 °C. The pellet was resuspended in cell culture media and stored at −80°C for further analysis.

### 2.5. sEV Characterization

sEV identity was confirmed by electronic microscopy and a nanoparticle tracking system, NanoSight NS300, as described previously [4].

### 2.6. Analysis of Pathways and miRNA Target Genes

In silico analysis of miR-302a-3p targets were performed using TargetScanHuman (https://www.targetscan.org/vert_80/; accessed on 4 September 2020) using Tarbase. The pathway analysis in which the detected miRNAs were involved was performed using DIANA TOOLS mirPath v.3 algorithm (http://snf-515788.vm.okeanos.grnet.gr/; accessed on 7 September 2020) [37]. Moreover, the analysis of the pathways regulated by the putative targets of the miRNAs was performed with the FUNRICH software using the gen ontology database. With STRING (https://string-db.org/; accessed on 20 September 2020), the networking of the targets was performed.

### 2.7. Transfection of miR-302a-3p Mimic

ARPE-19 cells were transfected with miR-302a-3p mirVana^®^ miRNA mimic (Thermo Fisher Scientific, Waltham, MA, USA), and mirVana™ miRNA mimic negative control #1 (Thermo Fisher Scientific, Waltham, MA, USA) as a negative control. The procedure of transfection of miRNA mimic and control was described previously [24]. After 48 h of transfection, ARPE-19 cells and cell culture media were collected for following analysis.

### 2.8. RNA Isolation from ARPE-19 Cells

Total RNA, including mRNA and miRNA, was isolated using an miRNeasy Mini Kit (Qiagen, Hilden, Germany), as described previously [24]. Total RNA was quantified by NanoDrop 2000 (Thermo Fisher Scientific, Waltham, MA, USA).

### 2.9. RNA Isolation from sEVs

RNA from sEVs was isolated using the commercial Total sEVs RNA and Protein Isolation Kit (Invitrogen, Thermo Fisher Scientific, Waltham, MA, USA) according to the manufacturer’s instruction. Total RNA was eluted with 50 µL of RNase-free water pre-warmed at 95 °C. The isolated RNA was stored at −80 °C. Quality and quantity of total RNA was measured by NanoDrop 2000 (Thermo Fisher Scientific, Waltham, MA, USA).

### 2.10. mRNA Expression Analysis

mRNA expression from ARPE-19 cells was carried out by quantitative real-time PCR (qRT-PCR). Retro transcription reaction of 1000 ng of RNA was performed with SuperScript III First-Strand Synthesis System (Life Technologies, Thermo Fisher Scientific, Waltham, MA, USA). For qPCR KiCq Start™ Primer (Sigma-Aldrich, St. Louis, MO, USA), Sybr Green Supermix (BioRad, Hercules, CA, USA), and RT-PCR Roche 234 LighterCycler 480 were used, with the appropriate temperature cycles as described previously [24]. The specific primers for VEGFA were: 5’-TGAAGGTCGGAGTCAACGGAT-3’ (forward) 5’-TTCTCAGCCTTGACGGTGCCA-3’ (reverse), and for GAPDH were: 5’-GACTTATACCGGGATTTCTTG-3’ (forward) 5’-AATGTGAATGCAGACCAAAG-3’ (reverse). GADPH was used as normalizer. Relative expression was calculated as 2^−∆∆Ct^.

### 2.11. miRNA Expression Analysis

qRT-PCR was performed to assess sEV miRNA expression levels. sEV RNA was retrotranscribed using a TaqMan MicroRNA Reverse Transcription Kit (Applied Biosystems, Bedford, MA, USA) using the specific TaqMan RT primer for miR-302a-3p. qPCR was performed using the TaqMan™ microRNA primer for miR-302a-3p (Thermo Fisher Scientific, Waltham, MA, USA) with TaqMan Gene Expression Master Mix (Applied Biosystems, Bedford, MA, USA) and RT-PCR Roche 234 LighterCycler 480, as described by Oltra et al. [24]. RNU6B snoRNA was used as normalizer. Relative expression was calculated as 2^−∆∆Ct^.

### 2.12. Statistical Analysis

Results of each experiment are presented as mean ± SEM. Each experiment was repeated at least three times. Statistical significance was set at 0.05 using a t-test or ANOVA followed by Tukey’s post hoc test, as appropriate. The statistical analysis was performed using GraphPad Prims 6 software.

## 3. Results

### 3.1. H_2_O_2_-Induced SEVs Promoted Oxidative Stress, Decreasing Cell Viability in ARPE-19 Cells

**Figure 1:**
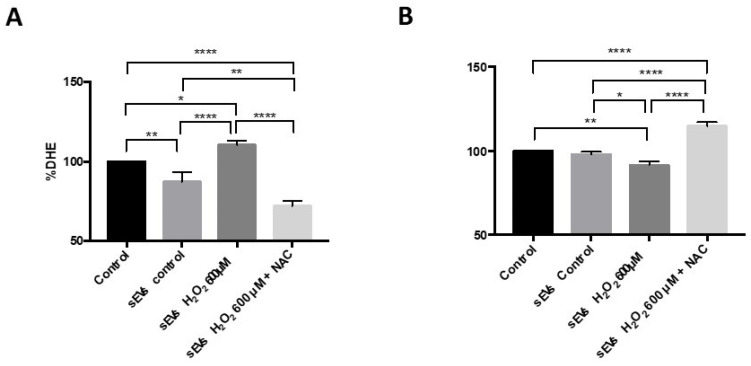
sEVs induced oxidative stress and affected cell viability. Superoxide anions were measured by DHE after sEV control (87.16 ± 6.23), sEV H_2_O_2_ 600 µM (110.5 ± 2.61), and sEV H_2_O_2_ 600 µM with N-acetylcysteine exposure (72.03 ± 6.61) (**A**). XTT assay was performed on the analyzed cell viability after sEV control (97.98 ± 1.58), sEV H_2_O_2_ 600 µM (91.38 ± 2.43), and sEV H_2_O_2_ 600 µM with N-acetylcysteine exposure (114.8 ± 2.31). (**B**). Values are expressed as percentage to control ± SEM (*n* = 4; DHE and *n* = 3–4; XTT). The *p*-value was calculated by ANOVA, and statistically significant differences were * *p* < 0.05, ** *p* < 0.01, and **** *p* < 0.0001.

**Figure 2:**
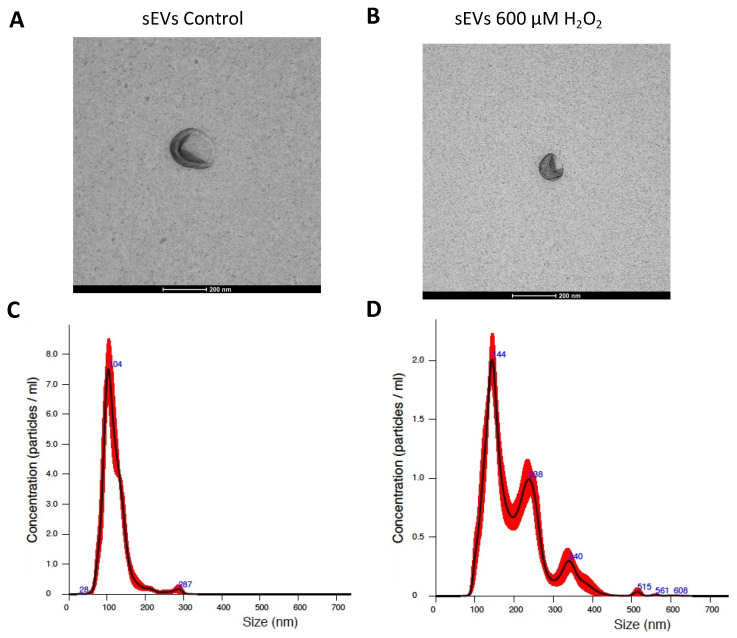
sEVs released by ARPE-19 cell characterization. sEVs released by control ARPE-19 cells (**A**) and 600 µM H_2_O_2_ ARPE-19 cells (**B**) were observed by electron microscopy and nanoparticle tracking analysis sEV control (**C**) and sEVs H_2_O_2_ 600 µM (**D**) Scale bar: 200 nm.

H_2_O_2_-induced SEVs (see the Materials and Methods section) resulted in significantly increased ROS levels (% DHE) when cultured with naïve ARPE-19 cells. This increase fitted with the observed ARPE-19 cell viability decrease. Both phenomena were significantly reduced by the addition of the antioxidant NAC. sEVs from control ARPE-19 cells resulted in significantly lesser oxidative levels compared to control (Figure 1A) and viability levels equal to control condition (Figure 1B). sEVs were observed using electronic microscopy and quantified by nanoparticle tracking system in order to characterized them (Figure 2).

### 3.2. miR-302a-3p Targets and Related Pathways

**Figure 3:**
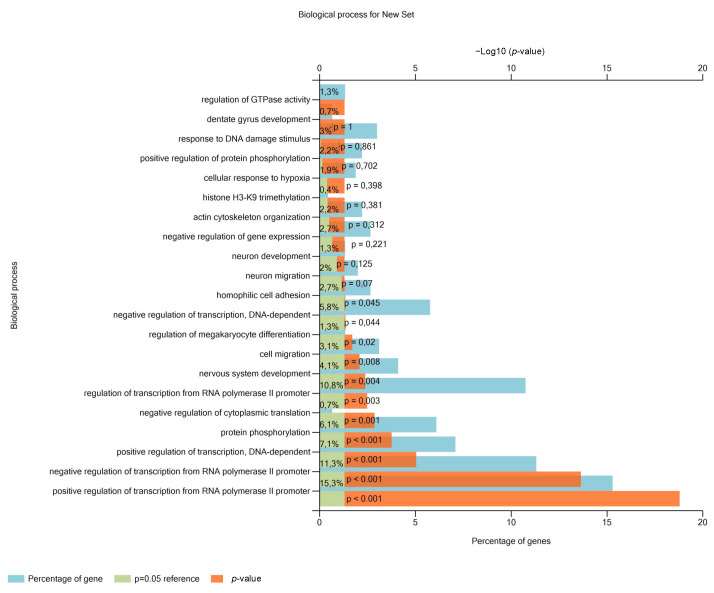
miR-302a-3p protein target-related pathways. Top 20 biological processes regulated by miR-302a-3p predicted targets, using FUNRICH and the Gene Ontology database.

Networks and biological pathways regulated by the predicted targets were entered into the FUNRICH software (version 3.1.3) to perform the functional analysis. The functional enrichment analysis of miR-302a-3p showed 607 biological processes regulated by at least one of the 1019 mRNA target of miR-302a-3p. Among the top 20 related pathways, 1.3% of genes were related to GTPase activity, dentate gyrus development by 0.7%, and DNA damage response by 3% (Figure 3).

**Figure 4:**
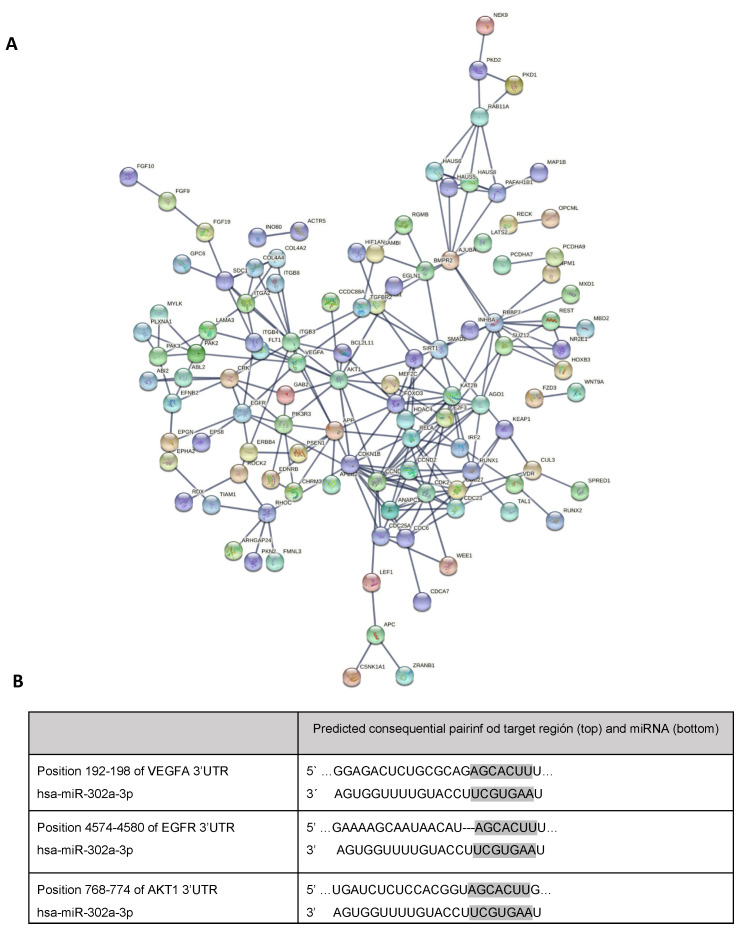
Angiogenic or vasculogenic pathways regulated by miR-302a-3p mRNA-related targets. Interactional network of miR-302a-3p angiogenic related targets, showing the connected ones (**A**). Predicted binding site of miR-302a-3p with the 3’UTR mRNA of VEGFA, EGFR, and AKT1 (**B**).

About 38 pathways related to miR-302a-3p mRNA targets are related to blood vessel formation. Using STRING, angiogenic and vasculogenic related processes were selected, and 213 mRNA predicted targets were found, which encode proteins involved in the regulation of angiogenic processes (Figure 4A). Among the most repeated mRNA targets related to vasculogenic processes are VEGFA, EGFR, and AKT1. In fact, in silico analysis showed that VEGFA, EGFR, and AKT1 mRNAs are predicted targets for miR-302a-3p (Figure 4B).

### 3.3. VEGFA mRNA and Angiogenesis Were Regulated by H_2_O_2_ and SEVs

**Figure 5:**
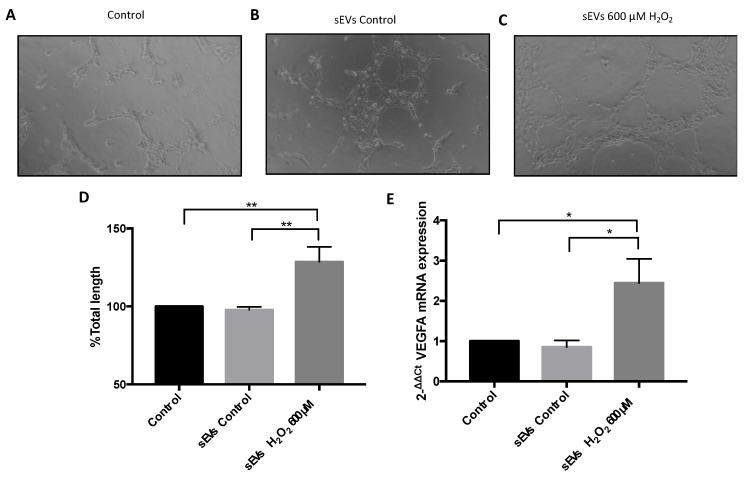
sEV-mediated vasculogenesis. HUVEC tube formation subject to different ARPE-19 cell culture media. Control medium (100 ± 0) (**A**), control sEVs (117.2 ± 1.79) (**B**), and sEV H_2_O_2_ 600 µM (128.7 ± 9.52) (**C**). Scale bar: 100 µM. Total length (**D**) was calculated. mRNA VEGFA expression after 24 h of sEVs control (0.85 ± 0.16) and sEVs H_2_O_2_ 600 µM (2.44 ± 0.6) exposure in ARPE-19 cells (**E**). Values are expressed as mean ± SEM (*n* = 3; Matrigel assay and *n* = 5; mRNA expression analysis). The *p*-value was obtained by ANOVA; * *p* < 0.05 and ** *p* < 0.01.

To assess the effect of sEVs on VEGFA mRNA expression, sEVs from both control and H_2_O_2_-treated ARPE-19 cells were isolated and cultured with naïve ARPE-19 cells. We previously reported that VEGFA mRNA was significantly overexpressed in ARPE-19 cells after H_2_O_2_ exposure [24]. Similarly, ARPE-19 cells cultured with H_2_O_2_-induced sEVs resulted in significantly increased VEGFA mRNA levels compared to control and control-induced sEVs (Figure 5E). In order to demonstrate the role of sEVs in angiogenesis, HUVEC cultured on standard cell culture medium or with sEVs from control ARPE-19 cells did not result in significant levels of angiogenesis (Figure 5A,B,D). However, cell culture media with H_2_O_2_-induced sEVs significantly increased angiogenesis in HUVEC cells (Figure 5C,D).

### 3.4. miR-302a-3p sEV Cargo Regulated Oxidative-Induced Angiogenesis

**Figure 6:**
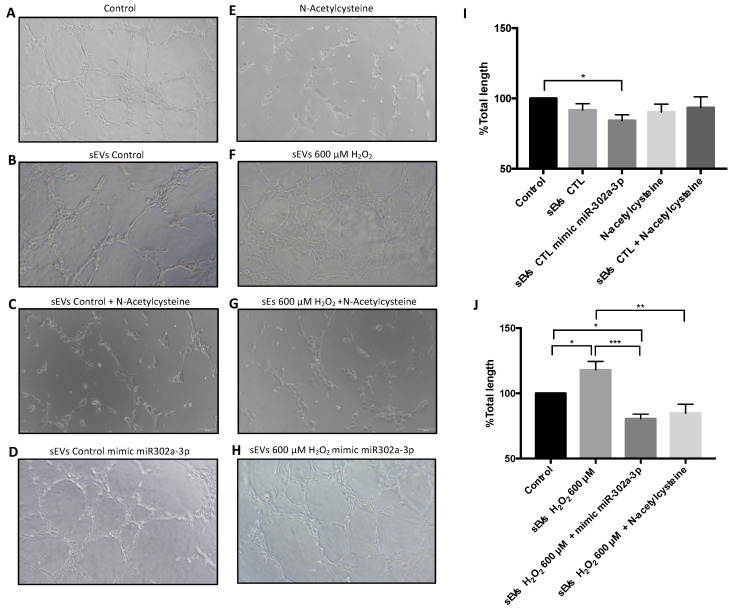
Angiogenesis was mediated by miR-302a-3p within sEVs. HUVEC tube formation subject to different sEVs released by ARPE-19 cell under different conditions. Control medium (100 ± 0) (**A**), sEVs released in control conditions (91,76 ± 4.5) (**B**), sEVs released in control conditions and N-acetylcysteine (93.44 ± 7.69) (**C**), sEVs released in control conditions with mimic miR-302a-3p (84.27 ± 4.15) (**D**), N-acetylcysteine (90.30 ± 5.61) (**E**), sEVs released in H_2_O_2_ 600 µM conditions (118 ± 6.43) (**F**), sEVs released in H_2_O_2_ 600 µM conditions and N-acetylcysteine (84.88 ± 6.79) (**G**), and sEVs released in H_2_O_2_ 600 µM conditions with mimic miR-302a-3p (80.57 ± 3.52) (**H**). Total length (**I**,**J**) was calculated. Values are expressed as mean ± SEM (*n* = 3). The *p*-value was calculated by ANOVA; * *p* < 0.05, ** *p* < 0.01, and *** *p* < 0.001.

HUVEC vasculogenic assay was performed to demonstrate the angiogenic effect of miR-302a-3p. HUVEC were grown with sEVs released from ARPE-19 cells under different conditions. Control-released sEVs keep the vascular processes at levels equals to fresh cell culture medium (Figure 6A,B,I). Moreover, sEVs released after miR-302a-3p mimic addition resulted in a significant vascular decrease (Figure 6D,I). As expected, H_2_O_2_-induced sEVs significantly increased angiogenic changes (Figure 6F,J). However, these changes were reduced by adding miR-302a-3p mimic (with H_2_O_2_ treatment, see the Materials and Methods section) (Figure 6G,J). All the aforementioned changes were normalized by NAC addition, indicating its oxidative nature (Figure 6C,E,G,I,J).

### 3.5. VEGFA mRNA Was Overexpressed by H_2_O_2_ and Modulated by miR-302a-3p

**Figure 7:**
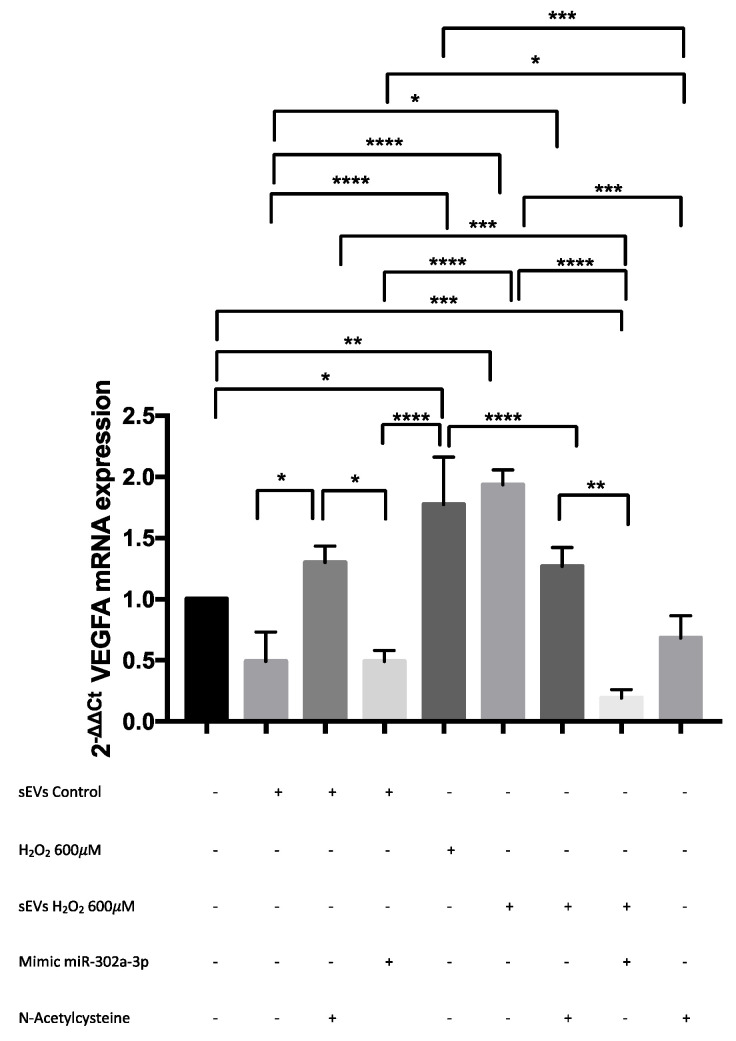
Proangiogenic molecules regulated by miR-302a-3p within sEVs. After 24h of sEV control (0.49 ± 0.24), sEV control and N-acetylcysteine (1.30 ± 0.14), sEV control and miR-302a-3p mimic (0.49 ± 0.09), H_2_O_2_ 600 µM (1.77 ± 0.39), sEVs H_2_O_2_ 600 µM (1.94 ± 0.12), sEVs H_2_O_2_ 600 µM and N-acetylcysteine (1.27 ± 0.16), sEV H_2_O_2_ 600 µM and miR-302a-3p mimic (0.19 ± 0.07), and N-acetylcysteine (0.68 ± 0.18) exposure the mRNA VEGFA expression was analyzed by q-RT-PCR. Values are expressed as mean ± SEM (*n* = 4–5). The *p*-value was calculated by ANOVA; * *p* < 0.05, ** *p* < 0.01, *** *p* < 0.001, and **** *p* < 0.0001.

In silico analysis indicated miR-302a-3p as a VEGFA target. ARPE-19 VEGFA mRNA expression was determined by qRT-PCR (Figure 7). VEGFA mRNA was significantly overexpressed by both H_2_O_2_ sEVs and H_2_O_2_ exposure compared to control sEVs and the control. miR-302a-3p mimic exposure significantly decreased the H_2_O_2_-induced VEGFA mRNA expression under the control mRNA expression levels. The addition of NAC significantly reduced H_2_O_2_-induced VEGFA mRNA expression to control levels.

**Figure 8:**
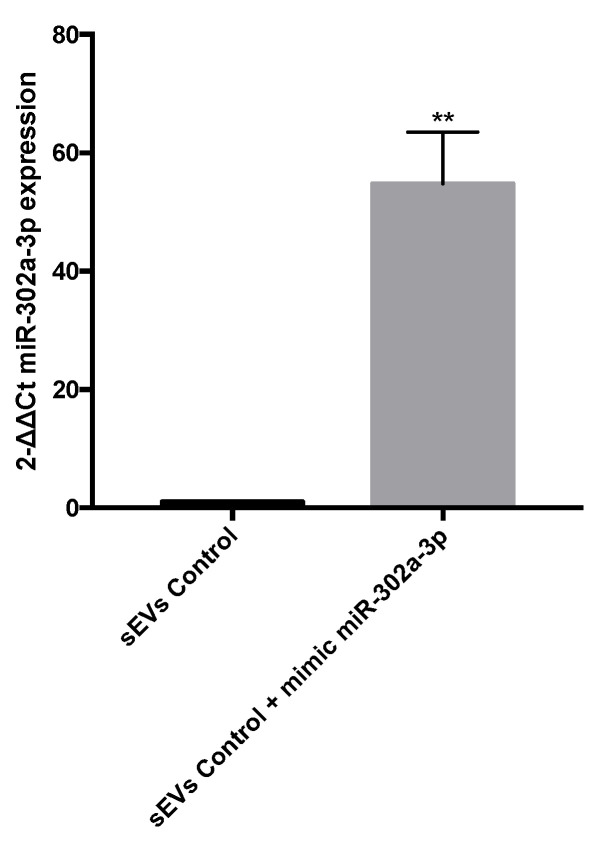
miR-302a-3p expression in control ARPE-19 cells. sEVs from untreated ARPE-19 cells (1 ± 0) and sEVs from miR-302a-3p mimic transfected ARPE-19 cells (54.8 ± 8.71). Values are expressed as mean ± SEM (*n* = 3). The *p*-value was calculated by t-test, and statistically significant differences were ** *p* < 0.01.

Finally, in order to demonstrate that miR-302a-3p mimic exposure is capable of affecting sEV cargo, we transfected ARPE-19 cells with miR-302a-3p mimic (see the Materials and Methods section), and sEVs were analyzed by qRT-PCR. Our findings strongly indicated that miR-302a-3p mimic transfection resulted in a significantly high miR-302a-3p expression levels on sEVs (Figure 8). This result supports the possibility of a direct targeting of miR-302a-3p on VEGF mRNA.

## 4. Discussion

The functional meaning of sEV release is currently gaining relevance on different physiopathological processes. It has been recently reported how oxidative-induced sEVs released from human RPE present Drussen-related proteins as part of the cargo, and this could be related to the pathological processes undergone in AMD patients [23]. Additionally, under hypoxia A431 cells release more than 50% of cytosolic proteins related to angiogenesis as part of the sEVs cargo [38]. We report herein how H_2_O_2_-induced sEVs can modulate angiogenesis via miR-302a-3p-VEGFA mRNA modulation.

One of the first noteworthy aspects is that H_2_O_2_-induced sEVs are capable of transmitting this “oxidative” condition. H_2_O_2_-induced sEVs increased ROS and decreased ARPE-19 cell viability. This fact matches with other studies that showed that the oxidative insult modulates the number of sEVs released and the nature of their cargo [1,4,39,40,41]. After seeing NAC results, one can argue that ROS or indirect pro-oxidant molecules are included as sEVs cargo. NAC was administered to ARPE-19 treated with H_2_O_2_-induced sEVs cultures, resulting in ROS level reduction. In addition, this process was accompanied by significantly decreased miR-302a-3p inside the sEVs [4]. Recently, we demonstrated how H_2_O_2_ stimulate angiogenesis processes [24]. Here, we demonstrate how the H_2_O_2_-induced sEVs can promote angiogenesis similarly to H_2_O_2_ exposure.

We previously reported how H_2_O_2_ promoted sEVs release on ARPE-19 cells containing significantly low miR-302a levels [4]. In agreement with others, we showed that miR-302a acts as a VEGFA mRNA modulator [33], and it has been shown that miR-302a-3p interacts with ZFP91, activating HIF-1 α (HIF-1 α is a classical VEGFA promoter) [34]. However, results herein indicate that miR-302a-3p directly interacts with VEGFA mRNA. This interaction could explain the vascular modification observed when adding miR-302a-3p mimic (Figure 6). On this line, and in agreement with our results, miR-302d and miR-302a were already considered as direct VEGFA mRNA modulators on HeLa and leukemia cells [42,43]. Similarly, VEGFA mRNA can also be directly modulated by miR-205 in glioblastoma cells or ARPE-19 cells [24,44]. These findings strongly suggest that different miRNAs can directly modulate VEGFA mRNA according to cell type origin such as miR-526b-3p, miR-150-5p, and miR-410, among others [45,46,47].

In order to demonstrate the role of miR-302a-3p in angiogenesis, we transfected ARPE-19 cells with a miR-302a-3p mimic on the different experimental conditions. The results indicate a marked negative regulation of miR-302a-3p on VEGFA mRNA expression levels/angiogenesis. H_2_O_2_-induced sEVs transfected with miR-302a-3p mimic resulted in reduced level of angiogenesis/VEGFA mRNA expression. This finding again fits with the suggestion of a direct miR-302a-3p/VEGFA mRNA binding as a mechanism of action. Interestingly, sEVs from H_2_O_2_-treated ARPE-19 cells significantly increase angiogenesis and VEGFA mRNA expression compared to control-treated cells. This agrees with previous data from our lab indicating that oxidative stress significantly decreases miR-302a-3p levels in sEVs, compared to control [4], thus providing support to the anti-angiogenic role of miR-302a-3p. Furthermore, it is well characterized that oxidative stress can promote the angiogenic drive [48,49,50]. VEGFA is upregulated by ROS through the PI3K/Akt pathway [51]. The addition of the antioxidant NAC to stressed condition normalized the increased VEGF mRNA/angiogenesis levels, suggesting it as an oxidative-dependent event.

In conclusion, oxidative stress promotes sEV release with pro-angionenic properties. The angiogenic effect is inhibited by miR-302a-3p and promoted by ROS. The mechanism of action seems to be related to the topological homology between VEGFA mRNA and miR-302a-3p. This opens a new strategy against oxidative-induced angiogenesis by promoting miR-302a-3p as a VEGFA mRNA repressor.

## Data Availability

Data is contained within the article.
